# The combined use of virtual reality and EEG to study language processing in naturalistic environments

**DOI:** 10.3758/s13428-017-0911-9

**Published:** 2017-05-26

**Authors:** Johanne Tromp, David Peeters, Antje S. Meyer, Peter Hagoort

**Affiliations:** 1 Max Planck Institute for Psycholinguistics, P.O. Box 310, 6500 AH Nijmegen, The Netherlands; 2 International Max Planck Research School for Language Sciences, Nijmegen, The Netherlands; 3 Donders Institute for Brain, Cognition and Behavior, Radboud University, Nijmegen, The Netherlands

**Keywords:** Language comprehension, Language processing, EEG, Virtual reality, N400

## Abstract

When we comprehend language, we often do this in rich settings where we can use many cues to understand what someone is saying. However, it has traditionally been difficult to design experiments with rich three-dimensional contexts that resemble our everyday environments, while maintaining control over the linguistic and nonlinguistic information that is available. Here we test the validity of combining electroencephalography (EEG) and virtual reality (VR) to overcome this problem. We recorded electrophysiological brain activity during language processing in a well-controlled three-dimensional virtual audiovisual environment. Participants were immersed in a virtual restaurant while wearing EEG equipment. In the restaurant, participants encountered virtual restaurant guests. Each guest was seated at a separate table with an object on it (e.g., a plate with salmon). The restaurant guest would then produce a sentence (e.g., “I just ordered this salmon.”). The noun in the spoken sentence could either match (“salmon”) or mismatch (“pasta”) the object on the table, creating a situation in which the auditory information was either appropriate or inappropriate in the visual context. We observed a reliable N400 effect as a consequence of the mismatch. This finding validates the combined use of VR and EEG as a tool to study the neurophysiological mechanisms of everyday language comprehension in rich, ecologically valid settings.

In everyday life, we often communicate about the things in our immediate environment. The information we can use to understand what someone is saying therefore often extends beyond words. For example, when visiting a restaurant we may listen to a friend talking about the food on her plate and the drinks on the table. We use visual information to understand what is being said. Consequently, realistic models of language comprehension should be able to explain language processing in this and many other types of contextually rich environments. Unfortunately, this is not always the case. In a recent overview, Knoeferle (2015) argued that psycholinguistic theorizing has been mostly “language-centric.” Most models (e.g., Bornkessel & Schlesewsky, 2006; Friederici, 2002) can explain a range of semantic and syntactic processes very well, but it is more difficult to derive hypotheses from them about how people comprehend language when they can use all sorts of information from the nonlinguistic environment (Knoeferle, 2015). One reason for the limited number of models with predictions on language processing in rich “real-life” contexts is that it is experimentally challenging to test them. It is difficult to design experiments with rich three-dimensional contexts that resemble our everyday environments, while maintaining control over the linguistic and nonlinguistic information that is provided. It becomes even more difficult if neurophysiological methods like electroencephalography (EEG) are used, which require strict control over the linguistic and nonlinguistic input and are sensitive to many nonrelevant signals from the environment. Here we test the validity of combining virtual reality (VR) and EEG to overcome this problem.

A virtual environment is a digital space in which sensory experiences are re-created and a user’s movements can be tracked (Fox, Arena, & Bailenson, 2009). VR can be used to create a three-dimensional world in which people can move and interact, which makes this paradigm a very suitable method to study psychological and social phenomena (Fox et al., 2009). By offering the possibility to re-create very complex, rich, everyday environments, VR allows researchers to increase the ecological validity of a study while maintaining full experimental control. This makes it possible to study behavior in different environments, without interference from uncontrollable cues, and allows for manipulations of variables that have traditionally been hard to replicate or control in the lab (Blascovich & Bailenson, 2011; Blascovich et al., 2002; Fox et al., 2009). Also, since virtual environments are often very engaging, they can be considered a motivational tool (Bayliss & Ballard, 2000). Finally, the use of virtual agents provides a good alternative to the use of human confederates, which is often problematic (Kuhlen & Brennan, 2013).

VR and EEG have been successfully combined, for instance, to study driving behavior (Bayliss & Ballard, 2000), spatial navigation (e.g., Bischof & Boulanger, 2003), and spatial presence (Baumgartner, Valko, Esslen, & Jänke, 2006). However, we are not aware of any studies that have combined VR and EEG to study language behavior. The reason for this might be the assumption that human–computer interactions are necessarily different from human–human interactions. This could be problematic if one wants to study everyday language behavior. However, recent evidence has suggested that this is not the case. In a study by Heyselaar, Hagoort, and Segaert (2017), participants performed the same syntactic-priming task with a human confederate and a human-like virtual agent and showed comparable priming effects in both situations. In addition, it has been shown that people adapt their speech rate and pitch to a virtual interlocutor in the same way that they do with a human interlocutor (Casasanto, Jasmin, & Casasanto, 2010; Gijssels, Casasanto, Jasmin, Hagoort, & Casasanto, 2016). Thus, VR has proven to be a useful tool to study language processes on a behavioral level. With the experiment proposed here, we hope to extend application of this technology to the neurophysiological level. As a proof of concept, we used VR and EEG to study language comprehension in an engaging visually rich three-dimensional environment. In particular, we investigated electrophysiological brain responses to mismatches between visual and auditory information.

In our experiment, people were immersed in a rich virtual environment (VE), a restaurant, while wearing EEG equipment. Several virtual agents (henceforth, “restaurant guests”) were seated at different tables in the restaurant, and participants were moved through the restaurant from table to table. Upon arrival, the participant looked at the object on the table in front of the guest (e.g., a plate with salmon), after which the guest produced a sentence (e.g., “I just ordered this *salmon*”). The noun in the sentence could either match (“*salmon*”) or mismatch (“*pasta*”) the object on the table, creating situations in which the auditory information was either appropriate or inappropriate with respect to the visual context. Thus, if successful, this setup would allow us to investigate electrophysiological brain activity during the simultaneous processing of auditory and visual information in a well-controlled, three-dimensional virtual environment.

Although not performed in VR, previous studies have used designs comparable to the one used here to investigate the neural correlates of language processing in an audiovisual context. For example, in a study by Peeters, Hagoort, and Özyürek (2015), participants viewed static pictures while they heard sentences that could either match or mismatch the information in the picture. For instance, participants saw a picture of a woman pointing at a mango while they heard a sentence that included either a matching noun (e.g., “I have just found this *mango* in the cupboard”) or a noun that did not match the visual information (e.g., “I have just found this *spoon* in the cupboard”). Incongruency between the spoken word and the physical object in the visual scene was reflected in an enhanced N400. The N400 is an event-related potential (ERP) component that peaks around 400 ms after the onset of a critical stimulus. The N400 has been linked to meaning processing and is sensitive to a wide variety of stimuli, including spoken and written words, objects, and sounds. Several theories exist concerning the functional significance of the N400 component (see Kutas & Federmeier, 2011, for an overview). One view is that the N400 reflects semantic integration (Brown & Hagoort, 1993; Hagoort, Baggio, & Willems, 2009), which is the process through which listeners use the global semantic representation from the context to immediately integrate the meaning of upcoming words into the overall message representation (Hagoort, 2003). In everyday language comprehension, the brain combines meaningful information from incoming speech with information about objects in the visual environment that are in the current focus of attention. Willems, Özyürek, and Hagoort (2008), for instance, investigated the neural integration of words and pictures into a preceding sentence context. In their ERP experiment, participants heard a word (e.g., “flower”) and saw a picture (e.g., of a flower) that had to be integrated with a preceding sentence context (e.g., “The man gave his wife a nice . . .”). The pictures and words could fit either well (e.g., flower) or less well (e.g., cherry) with the previous sentence context. If the item presented did not match the previous sentence context well, an N400 effect was observed. This effect was very similar for pictures and words in terms of latency and amplitude, suggesting that no differentiation between verbal and visual semantic information was made at this level of processing. In addition, an effect in an earlier time window (225–325 ms) was also not specific to the picture or word condition (Willems et al., 2008).

In addition to pictures, researchers have used videos to provide visual context to investigate semantic processing in more real-world environments (e.g., Sitnikova, Kuperberg, & Holcomb, 2003; Sitnikova, West, Kuperberg, & Holcomb, 2006). Sitnikova, Holcomb, Kiyonaga, and Kuperberg (2008) presented participants with movie clips of everyday events (e.g., cutting bread). The clips consisted of a context (e.g., a man placing a cutting board on a kitchen counter and then placing a loaf of bread on the cutting board) and a final scene. The final scene could match the previous scene (e.g., the man cuts off a piece of bread with a knife), violate the goal-related action requirements (e.g., the man slides an electric iron across the loaf of bread), or be completely unexpected (e.g., the man uses an electric iron to press wrinkles from his pants). Importantly, both mismatch conditions resulted in larger N400s than the match condition. Furthermore, an early semantic congruency effect was observed in the N300 window (250–350 ms). The authors suggested that this N300 effect reflected the fast access that visual images have to semantic memory networks (see also McPherson & Holcomb, 1999; Sitnikova et al., 2006). Finally, when the goal-related action requirement was violated (i.e., the ironing scene), a posterior late positivity was observed (Sitnikova et al., 2008). Although this experiment did not investigate the integration of visual and auditory information, since the violations occurred within the visual domain, the results offer predictions as to the latencies and distribution of ERP effects when participants are looking at a nonstatic environment.

In the field of gesture and sign language research, the use of videos is common, since semantic processing here critically hinges on the visual information provided (Andric & Small, 2012; Dick, Mok, Beharelle, Goldin-Meadow, & Small, 2014; Özyürek, 2014). For example, Özyürek, Willems, Kita, and Hagoort (2007) investigated the online integration of semantic information from speech and gesture. Participants listened to sentences with a critical verb (e.g., “He slips on the roof and *rolls* down”), combined with a video of an iconic gesture (e.g., a rolling gesture). The verbal and/or gestural semantic content could either match (“rolls” and a rolling gesture) or mismatch (“walks” and a walking gesture) the part of the sentence before the critical verb (e.g., “He slips of the roof and . . .”). The results revealed effects in the N400 window for both gestural and spoken mismatches, suggesting that information from both modalities is integrated at the same time.

Although the use of videos to study language comprehension in context is already a step away from using static pictures on a computer screen, it still has certain limitations that could be overcome by exploiting recent advances in VR technology. First, videos provide only a two-dimensional scene on a very small computer screen, whereas in VR participants experience a very large, realistic, three-dimensional environment. Furthermore, in VR it is possible for participants to look at a dynamic speaker and even interact with him or her, rather than just observe a person on a screen. Recently it has been argued that to study the brain basis of interaction, we should move away from passive spectator science to studies with engaged participants (Hari, Henriksson, Malinen, & Parkkonen, 2015). VR is a useful method to do so, provided that reliable effects can be observed in an environment that is much more complex and dynamic, but also more distracting, than a simple computer screen. With the experiment described here, we aimed to test the feasibility of combining VR and EEG to study language comprehension in a rich setting. On the basis of the studies mentioned above, we predicted an N400 effect for our study as well. The amplitude in the N400 window should be more negative for the noun in the mismatch condition (e.g., “I just ordered this *pasta*” when a piece of *salmon* is on the table) than in the match condition (e.g., “I just ordered this *salmon*” when a piece of *salmon* is on the table). Finding an N400 effect would validate the combined use of VR and EEG as a tool to study everyday language comprehension in rich, ecologically valid settings, thereby paving the way for future experimental studies of the neurophysiological mechanisms involved in everyday language use.

## Method

### Participants

Twenty-three participants (21 females, two males) with an average age of 21 years (range 18–26) participated in the experiment. All were right-handed native speakers of Dutch, had normal or corrected-to-normal vision and normal hearing, and had no history of speech problems or neurological diseases. Participants provided written informed consent and were paid to participate in the experiment. Ethical approval for the study was granted by the ethics board of the Social Sciences Faculty of Radboud University. Two participants were excluded from the analysis due to technical failures during the experiment. The data from one additional participant were excluded because too many trials (>30% per condition) had to be discarded due to EEG artifacts.

### Materials and design

**Fig. 1:**
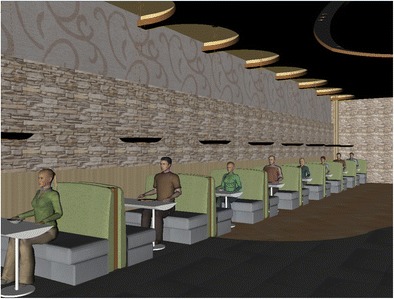
Screenshot of the virtual environment

The experiment took place in a virtual environment (VE) that was custom-made using Vizard (version 4.08; WorldViz, Santa Barbara, CA). It consisted of a restaurant with eight tables in one row and a virtual restaurant guest sitting at each table (see Fig. 1).

Participants were passively moved from table to table through the restaurant via a preprogrammed procedure (i.e., they did not physically walk themselves). This procedure was chosen to reduce movement artifacts in the EEG data and to control the amount of time that a participant was able to look at the object on each table. All of the restaurant guests resembled Caucasian males or females between the ages of 25 and 35, in line with the age, gender, and background of the speakers who recorded the sentences. The restaurant guests kept a neutral facial expression throughout the experiment. The voices of the virtual guests were rendered with a stereo speaker set.

The materials consisted of 80 objects and 96 sentences. There were 80 experimental sentences and 16 fillers. On each trial, participants saw an object on the table in the VE (see below) and then heard a sentence from a restaurant guest seated at the table. All of the sentences and objects were relevant to a restaurant setting. The sentences (e.g., the Dutch equivalent of “I just ordered this salmon.”) were paired with objects (e.g., a plate with salmon) so that the critical noun in the sentence could either match (e.g., “salmon”) or mismatch (e.g., “pasta”) the object on the table. The determiner preceding the noun always matched both the gender of the noun corresponding to the object on the table and the noun spoken by the virtual agent (which differed in the mismatch condition). The filler sentences were general statements that could be uttered in a restaurant setting but did not refer specifically to an object in the VE (e.g., “I always come here for lunch”). During presentation of the filler sentences, a generic cup, plate, or bowl was visible on the table. The sentences were recorded by eight native speakers of Dutch (four male, four female), had an average duration of 1,973 ms (*SD* = 354), and were equalized in maximum amplitude using the speech analysis package Praat (version 5.1; www.praat.org). The onset of the critical noun was determined in Praat. The experimental sentences had ten different sentence frames (e.g., “*Ik heb deze . . . net besteld*,” “I have just ordered this . . .”)*.* Each speaker used each sentence frame only once, and each frame was presented in each round (or block) only once. Half of the sentences were presented in the match condition and half in the mismatch condition, counterbalanced across participants, which resulted in two lists. Sentences were never repeated for a participant. The objects were each repeated once, with a minimum of 32 trials (four blocks) between two presentations of the same object.

### Procedure

Participants were seated in a chair while they wore an EEG cap beneath an NVIS nVisor SX60 head-mounted display. The display presented the VE at a 1,280 × 1,024 resolution, with a 60-deg monocular field of view. Eight reflective markers were mounted onto the head-mounted display, which were linked to a passive infrared DTrack 2 motion-tracking system (ART Tracking, Munich). The data from this system were used to update the participant’s viewpoint when the participant moved.

Prior to entering the VE, participants were told that they would move through a restaurant and that the guests in the restaurant would say something to them. Participants were instructed to pay close attention to the objects on the tables and to what the restaurant guests said. To familiarize participants with the food and drinks served in the virtual restaurant, they were asked to look at the menu of the restaurant prior to the start of the experiment, which contained all of the objects, and their labels, that could be presented in the VE.

The trial sequence was as follows: From the beginning of a trial, participants “arrived” at the table in 2 s (i.e., the movement took 2 s). Upon arrival, the participant had 4 s to look at the object on the table. Then the restaurant guest looked up, and 2 s later he or she began to speak. At the end of the sentence, the participant was moved backward again automatically. Before the start of the experiment, participants were instructed to keep eye contact with the restaurant guest from the moment the guest looked up to the end of the sentence. They were also encouraged not to blink their eyes during this period.

Participants made 12 rounds through the restaurant. During each round, each restaurant guest said one sentence, resulting in eight sentences per round. After each round, participants were encouraged to take a short break. Before the first experimental round, the participant completed a practice round in which they were moved past each table. During this round, participants could get used to the movement and were encouraged to practice looking up at the restaurant guest and not blinking while making eye contact with him or her. There were no objects on the tables during the practice round, and the restaurant guests only looked up and did not speak.

After the experiment participants were asked to complete two questionnaires. The first evaluated whether they had paid attention during the experiment. It contained eight statements: four about the sentences (e.g., “An avatar said that he/she always comes here for breakfast.”) and four about the objects (e.g., “One of the objects in the restaurant was a pear.”). Participants were asked to choose “true,” “false,” or “I don’t know.” The percentage of correct responses was calculated on the basis of the “true” and “false” responses. If participants filled in “I don’t know” (5.00% for the object questions, 3.75% for the sentence questions), this was not counted as a response. The aim of the second questionnaire was to assess the participant’s perceptions of the virtual agents. The questionnaire consisted of eight questions about the appearance and behavior of the restaurant guests (e.g., “How human-like did you find the avatars?”). Participants were asked to response on a scale from 1 (*not human-like*) to 7 (*very human-like*).

### EEG recording and analysis

**Fig. 2:**
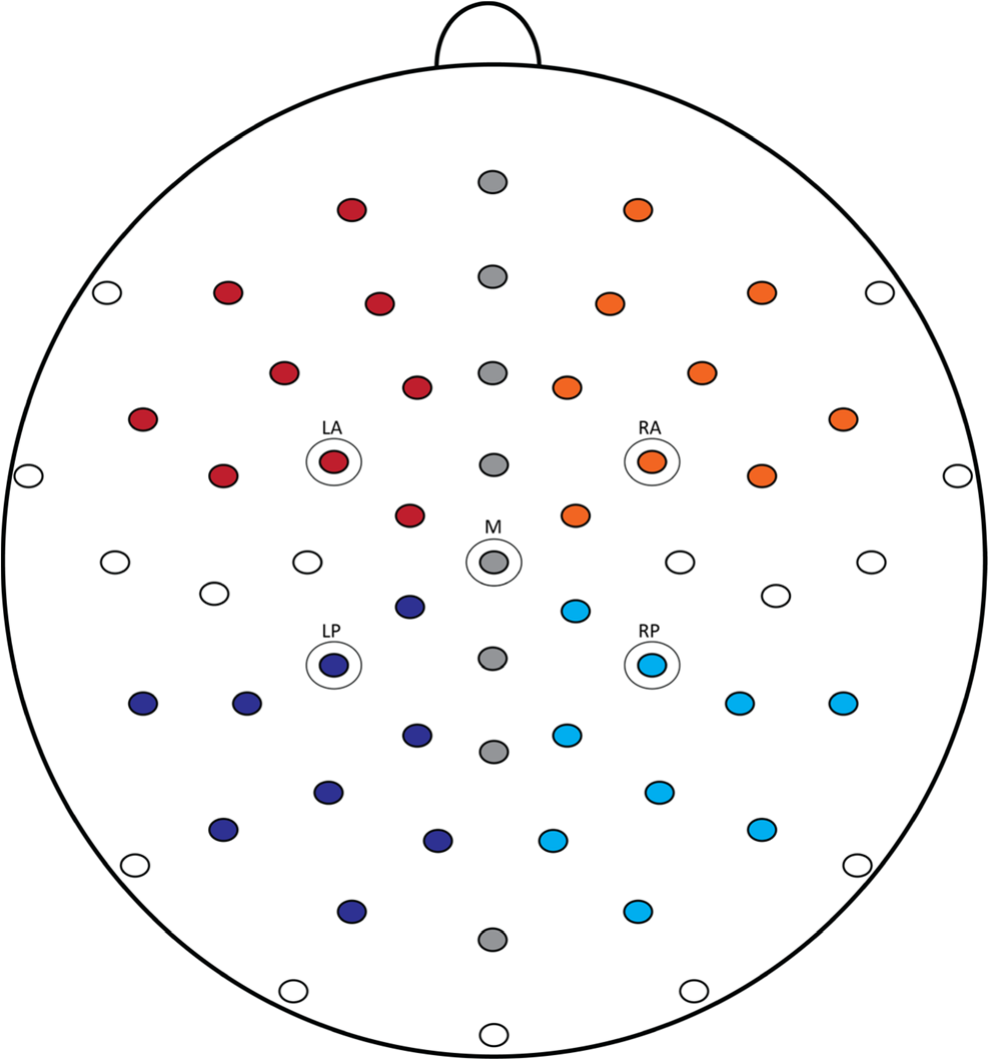
Equidistant electrode montage. The electrode sites displayed in Fig. 3 are circled (LA, left anterior; RA, right anterior; M, midline; LP, left posterior; RP, right posterior). The five regions used in the analysis are highlighted in different colors (LA = red; RA = orange; M = gray; LP = dark blue; RP = light blue)

The electroencephalogram (EEG) was continuously recorded from 59 active electrodes held in place by an elastic cap (see Fig. 2 for the equidistant electrode montage). In addition to the electrodes in the cap, three external electrodes were attached: one below the left eye, to monitor for blinks, and one on the lateral canthus to the side of each eye, to monitor for horizontal eye movements. Finally, two electrodes were placed over the left and the right mastoid, respectively. The electrodes were referenced online to the electrode placed over the left mastoid, and offline to the average of the left and right mastoids. Electrode impedance was kept below 20 kΩ. The EEG was recorded with a low cutoff filter of 0.01 Hz and a high cutoff filter of 200 Hz at a sampling rate of 500 Hz. A high-pass filter at 0.01 Hz and a low-pass filter at 40 Hz were applied offline. The Brain Vision Analyser software (Version 2.0.2, Brain Products, Munich) was used to process the EEG. Epochs from 100 ms preceding the onset of the critical noun to 1,200 ms after the critical noun were selected. Trials containing ocular artifacts were excluded (8.88% in the match condition, 9.63% in the mismatch condition; not statistically different). The 100-ms period preceding the critical noun was used as a baseline. Average ERPs were calculated per participant and condition in three time windows. In addition to the N400 window (350–600 ms), an earlier window (250–350 ms) was included, in light of previous studies that had observed early effects as a result of visual or audiovisual mismatches (e.g., Peeters et al., 2015; Sitnikova et al., 2008; Willems et al., 2008). Finally, a 200-ms window after the N400 window was analyzed (600–800 ms) to test for the presence of a sustained N400 effect. Repeated measures analyses of variance (ANOVAs) were performed in the different time windows with the factors condition (match, mismatch), region (vertical midline, left anterior, right anterior, left posterior, left anterior), and electrode. The Greenhouse–Geisser correction (Greenhouse & Geisser, 1959) was applied to all analyses with more than one degree of freedom in the numerator; the adjusted values are reported.

## Results

On average, participants answered 86.46% (*SE* = 0.90%) of the questions correctly in the attention questionnaire. They scored 77.92% (*SE* = 4.54%) on the object questions (e.g., “One of the objects in the restaurant was a pear.”) and 95.00% (*SE* = 2.29%) on the questions about the sentences (e.g., “An avatar said that he/she always comes here for breakfast”). The results from the second questionnaire indicated that the restaurant guests were rated as relatively human-like (*M* = 4.6, *SE* = 0.06).

**Fig. 3:**
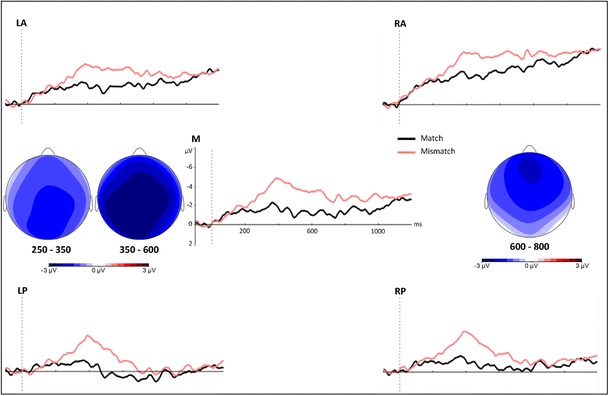
Grand-average waveforms time-locked to the onset of the critical nouns in the match and mismatch conditions. The topographic plots display the voltage differences between the two conditions (mismatch – match) in the three different time windows

Figure 3 displays the grand average waveforms time-locked to the onset of the critical noun. The ANOVA for the early time window (250–350 ms) revealed a significant main effect of condition [*F*(1, 19) = 6.22, *p* = .03, *η*_p_^2^ = .25]. ERPs were more negative for the mismatch condition (*M* = –2.42 *μ*V, *SE* = 0.32) than for the match condition (*M* = –1.34 *μ*V, *SE* = 0.46). This effect was not modulated by region (*F* < 2).

In the N400 window (350–600 ms), we also found a significant main effect of condition [*F*(1, 19) = 18.03, *p* = .001, *η*_p_^2^ = .49], with a more negative ERP for the mismatch condition (*M* = –2.99 *μ*V, *SE* = 0.42) than for the match condition (*M* = –1.30 *μ*V, *SE* = 0.58). The effect was widespread, confirmed by the absence of a Condition × Region interaction (*F* < 2).

Visual inspection of the waveforms in Fig. 3 indicates a continuation of the N400 effect in the 200-ms epoch right after the standard N400 window (600–800 ms). This observation was confirmed by the ANOVA for this window, which revealed a main effect of condition [*F*(1, 19) = 10.10, *p* < .01, *η*_p_^2^ = .35]. The amplitude once again was more negative for the mismatch condition (*M* = –2.33 *μ*V, *SE* = 0.67) than for the match condition (*M* = –1.23 *μ*V, *SE* = 0.61). This effect was not modulated by region (*F* < 3).

## Discussion

The aim of this study was to test the validity of the combined use of VR and EEG to study language comprehension in a visually rich context. Participants were immersed in a virtual environment, a restaurant, in which the virtual restaurant guests were seated at tables with food or drinks in front of them. The guests produced sentences that could match (e.g., “I just ordered this salmon”) or mismatch (e.g., “I just ordered this pasta”) the object on the table before them (e.g., a piece of salmon). As a result of this manipulation, we observed a reliable N400 effect, in line with our predictions. This shows that VR and EEG combined can be used to study language comprehension in realistic three-dimensional environments. Neither the VR helmet (head-mounted display) placed over the EEG cap nor the noise caused by the VR equipment limited us in acquiring a reliable EEG signal. There were also not more artifacts, due to movement or blinking, than in an average EEG study. The rich virtual environment was not too distracting for the participants, since they paid attention to the restaurant guests and objects and judged the restaurant guests to be human-like. It should be noted that the percentage of correct answers in the attention questionnaire was lower for the objects than for the sentences, which might suggest that participants did not pay enough attention to the objects. We believe, however, that this difference was due to the fact that participants were presented with the menu of the restaurant, which contained all of the objects, prior to seeing a subset of the objects in the actual experiment. Thus, they might have remembered objects from the menu rather than from the experiment itself, which resulted in the higher percentage of errors.

In all time windows, ERPs were more negative for the mismatch than for the match condition. Importantly, in the N400 window there was a widely distributed, ongoing negativity similar in onset latency and distribution to the effects observed in previous studies that had investigated the integration of visual and auditory information (e.g., Peeters et al., 2015; Willems et al., 2008). This negativity extended into the 600- to 800-ms time window. The extended nature of the N400 effect in our study could simply be a carryover from the strong N400 effect (e.g., Willems et al., 2008), or it could reflect the extended presentation time of the incongruous information. In our study, participants were able to see the object even after the restaurant guest had already stopped speaking, which resulted in a more prolonged negativity than the typical N400 effect evoked by short presentation of written or spoken words (Sitnikova et al., 2008). Finally, ERPs were also more negative for the mismatch than for the match condition in an early time window (250–350 ms). In Sitnikova et al. (2008), the negativity in this window was interpreted as a separate N300 effect, reflecting the rapid access to visual information within semantic memory networks. However, since in our study the mismatching information came from the speech signal (in the context of visual information), it is unlikely that this account would hold for the present data. Rather, the effect resembles early effects observed in other studies investigating mismatches in auditory speech processing (e.g., Connolly & Phillips, 1994; Hagoort & Brown, 2000). In these studies it has been suggested that a negativity in this window is an indication of a mismatch between the expected word forms, based on the context, and the activated lexical candidates generated on the basis of the speech signal (a phonological mismatch negativity; Connolly & Phillips, 1994). In our experiment, participants could build up a strong expectation or prediction for the word form of the upcoming noun on the basis of the visual context (i.e., they saw the object on the table well in advance). In addition, for most of the stimuli a mismatch could already be detected during the first segment of the noun (in 98.96% of our item sets, the onset of the mismatching noun was different from the word form expected on the basis of the visual context). Thus, it is very probable that the negativity observed in the early window (250–350 ms) was due to a mismatch between the expected and encountered word forms.

Although the present study was successful in providing evidence for the reliability of the combined use of VR and EEG, it has certain limitations. First, in a few cases there was some difficulty in setting up the EEG cap and VR helmet. The head-mounted display used in this study was meant to fit relatively tightly around the head, which in some instances made it somewhat challenging to use it in combination with an EEG. More recently developed head-mounted displays (e.g., the Oculus Rift) are lighter and more flexible than the one used in the present study, which will allow for longer experiments and reduced EEG preparation time preceding the start of the experiment. Moreover, the limitations of head-mounted displays can easily be overcome by using VR equipment (such as a CAVE system) that does not necessarily make use of a head-mounted display, but instead has participants wear 3-D shutter glasses to experience immersion in a VE. Finally, because of the combination with EEG, the VE could not be used to its full potential. In real life, people move their head, look around, and interact with the environment, which is all possible in VR as well. However, in our experiment such behavior was restricted because of the sensitivity of EEG to movement artifacts.

The combination of VR and EEG has the potential to address several underresearched questions in the field of psycholinguistics and the neurobiology of language. It can be used to study how we comprehend language when we use multiple sources of information in our environment, which is necessary for the development of more complete models of language processing (Knoeferle, 2015). Also, it can shed light on how we listen and speak in interactive real-world situations. The need for a shift away from spectator science and toward more interactive and realistic paradigms to study the human brain and human behavior has also been echoed in other fields of neuroscience. Social interaction plays a central role in human brain function, and it has been argued that studies in social neuroscience should shift their focus toward the inclusion of engaged participants and dynamic stimuli (Hari et al., 2015; see also Willems, 2015). Along similar lines, Schilbach and colleagues (2013) highlighted the necessity of studying real-time social encounters in an interactive manner. VR is well-suited to help us understand how we interact with others (virtual agents, avatars, or humans) during real-time communication. Research into the electrophysiology of language comprehension has been virtually “speakerless,” which has left the social, pragmatic, and dynamic functions of communication severely underresearched (Hoeks & Brouwer, 2014). VR provides a way to include a well-controlled speaker in our experiments, to study aspects of language and communication in a more natural, dynamic way, even in combination with electrophysiological recordings.
